# Proteoglycan Expression in Normal Human Prostate Tissue and Prostate Cancer

**DOI:** 10.1155/2013/680136

**Published:** 2013-04-18

**Authors:** Anastasia V. Suhovskih, Lyudmila A. Mostovich, Igor S. Kunin, Mekhrozhiddin M. Boboev, Galina I. Nepomnyashchikh, Svetlana V. Aidagulova, Elvira V. Grigorieva

**Affiliations:** ^1^Institute of Molecular Biology and Biophysics SB RAMS, Timakova Street 2, Novosibirsk 630117, Russia; ^2^MTC, Karolinska Institute, Nobels vag 16, 171 77 Stockholm, Sweden; ^3^Novosibirsk State Medical University, Krasnii Prospect 52, Novosibirsk 630091, Russia; ^4^Central Regional Hospital, Zalesskogo Street 6, Novosibirsk 630047, Russia; ^5^Institute of Regional Pathology and Pathomorphology SB RAMS, Timakova Street 2, Novosibirsk 630117, Russia

## Abstract

Proteoglycans (PGs) are expressed on the cell surface and extracellular matrix of all mammalian cells and tissues, playing an important role in cell-cell and cell-matrix interactions and signaling. Changes in the expression and functional properties of individual PGs in prostate cancer are shown, although common patterns of PGs expression in normal and tumour prostate tissues remain unknown. In this study, expression of cell surface and stromal proteoglycans (glypican-1, perlecan, syndecan-1, aggrecan, versican, NG2, brevican, decorin, and lumican) in normal tissue and prostate tumours was determined by RT-PCR analysis and immunostaining with core protein- and GAG-specific antibodies. In normal human prostate tissue, versican, decorin, and biglycan were predominant proteoglycans localised in tissue stroma, and syndecan-1 and glypican-1 were expressed mainly by epithelial cells. In prostate tumours, complex changes in proteoglycans occur, with a common trend towards decrease of decorin and lumican expression, overall increase of syndecan-1 and glypican-1 expression in tumour stroma along with its disappearance in tumour epithelial cells, and aggrecan and NG2 expressions in some prostate tumours. All the changes result in the highly individual proteoglycan expression patterns in different prostate tumours, which may be potentially useful as molecular markers for prostate cancer personalised diagnosis and treatment.

## 1. Introduction

Prostate cancer is a second leading cause of cancer dearth for men over the world. Study for the molecular mechanisms of prostate carcinogenesis is of basic importance for the development of new strategies for prostate cancer treatment.

According recent data, both cancer cells and tumour microenvironment coevolution contribute to the malignant transformation [1, 2]. And a special role is attributed to the molecules presented at both locations, such as proteoglycans, key molecular effectors of cell surface, and pericellular microenvironment [3, 4]. Progressive changes in cell surface and tissue stroma proteoglycans occur in different cancers including prostate cancer. 

The most studied proteoglycans in prostate cancer include extracellular proteoglycans versican, decorin and perlecan, and cell surface proteoglycans syndecan-1 and betaglycan [5].

It was shown that versican is overexpressed in benign prostate hyperplasia (BPH) and prostate cancer, where it is accumulated in the stromal compartment and potentially contributes to disease pathology [6]. High versican concentration was associated with increased risk of prostate-specific antigen (PSA) progression [7].

Extracellular antiproliferative proteoglycan decorin shows reduced expression in prostate cancer stroma compared to nonmalignant prostate stroma [8]. Systemic delivery of decorin to prostate-specific Pten(P^−/−^) mice, a genetically defined, immune-competent mouse model of prostate cancer, inhibits tumour progression by targeting cell proliferation and survival pathways [9].

High perlecan expression in prostate cancer cell lines and prostate cancer tissues correlates with a high Gleason score and rapid cell proliferation, and inhibition of perlecan expression in prostate cancer cell lines decreases cell growth [10]. Inhibitors of perlecan function at either the protein or glycan level were suggested as ideal drug candidates for anticancer therapies [11].

Controversial data are shown for cell surface heparan sulfate proteoglycan syndecan-1. 

From the one side, syndecan-1 overexpression in prostate tumours was significantly associated with early recurrence, tumour specific survival and high Gleason grade [12], with established features of biologically aggressive prostate cancer [13] and poor survival [14]. All the data suggested the expression of syndecan-1 as a prognostic marker for patients with clinically localised prostate cancer. 

From the other side, decreased syndecan-1 expression was shown in the prostate cancer cell lines LNCaP, PC3, and DU145 compared with the normal prostate epithelial cells [15]. Among the cell lines, syndecan-1 expression was much higher in the androgen independent prostate cancer cell lines DU145 and PC3, rather than the androgen-dependent LNCaP, suggesting that syndecan-1 might participate in the process of androgen-dependent to -independent conversion [16]. Immunohistochemical analysis revealed intensive syndecan-1 staining in normal prostate glands, whereas the expression was significantly decreased in prostate cancer samples [16]. Benign glands also showed significantly higher intensity staining for syndecan-1 than localised prostate cancer, and no significant association between syndecan-1 expression in prostate tumours and any of the following: Gleason score, pathological stage, surgical margin status, and biochemical recurrence was shown, suggesting that syndecan-1 is not an independent predictor of recurrence or tumour-specific survival and diminishing its significance as a clinical marker [17]. 

Some other proteoglycans such as aggrecan, neurocan, brevican, biglycan, lumican, glypicans, fibromodulin, agrin, collagen XVIII, NG2, and serglycin are also involved in cancer progression, but their expression and functional role have not yet been investigated in prostate cancer.

Evidently, there are both pro- [7, 11, 13] and antitumourigenic [8, 9] proteoglycans, and their combination in normal or cancer tissue could be of principal importance for normal tissue physiology and pathological transformation. However, most of the published studies were done for the individual proteoglycans, and no research on the simultaneous detection of different proteoglycans expressions in the same cells or tissues has been done.

In this study, we aimed to perform a comparative analysis for proteoglycans expression patterns in normal human prostate tissue, benign prostate hyperplasia, and prostate tumours *in vivo*. Expression of different proteoglycans (versican, decorin, perlecan, syndecan-1, glypican-1, aggrecan, NG2, brevican, and lumican) was determined both at protein and glycosaminoglycan levels using a combination of protein core and glycosaminoglycan-specific antibodies.

## 2. Materials and Methods

### 2.1. Materials and Antibodies

Mouse monoclonal antihuman syndecan-1, rabbit polyclonal antihuman glypican-1, and mouse monoclonal antihuman beta-actin antibodies were obtained from Abcam (UK); rabbit polyclonal antihuman lumican, mouse monoclonal antihuman decorin, and mouse monoclonal antihuman HS antibodies were obtained from Abnova (USA); mouse monoclonal antihuman CS antibody was purchased from Sigma (USA); FITC-conjugated antibody against mouse IgGs and TexasRed-conjugated antibody against rabbit IgGs were obtained from Vector Laboratories (USA). ProLong Gold antifade reagent with DAPI was purchased from Invitrogen (USA).

### 2.2. Patients and Tissue Samples

All tissue samples were obtained from benign prostate hyperplasia (BPH) and primary prostate tumours during radical surgery at the Central Municipal Hospital N1 and Central Region Hospital, Novosibirsk, Russia. Tissues were collected into RNALater solution (Ambion, USA) and stored at −20°C. Tumour regions were manually dissected from the blocks to provide a consistent tumour cell content of more than 70% for analysis. The prevalent histological type of the tumours was adenocarcinoma, with different degrees of malignancy. Most patients were at the second stage of malignancy progression according to the TNM formula. PSA was 1,26–5,9 ng/mL for BPH and 7,5–33 ng/mL for adenocarcinoma patients, Gleason scores 5–7. Totally, 18 clinical samples were analysed. All patients provided written informed consent, and the study protocol was approved by the Local Ethics Committee in accordance with the Helsinki Declaration of 1975. 

### 2.3. Total RNA Isolation

Total RNA was extracted from the tissue samples using TRIzol Reagent (Invitrogen, Carlsbad, CA, USA) according to the manufacturer's instructions. DNA contamination was removed using TURBO DNA-*free* kit (Ambion) according to the manufacturer's instructions. The integrity and quality of the isolated RNA were checked by agarose gel electrophoresis, and total RNA concentration was measured with Qubit-iT RNA Assays Kit (Invitrogen) according to the manufacturer's instructions.

### 2.4. Multiplex RT-PCR

cDNA was synthesised from 1-2 *μ*g of total RNA using a First Strand cDNA Synthesis Kit (Fermentas, Hanover, MD, USA), and 1/10th of the product was subjected to PCR analysis. 

The following conditions were used for multiplex RT-PCR: 95°C for 5 min, 95°C for 30 sec, 55–63°C for 30 sec, and 72°C for 1 min, with a final elongation step at 72°C for 10 min using a Piko Thermal Cycler (Fisher Scientific, USA). The total reaction volume was 10 *μ*L. The PCR primers and conditions used were from [18] and listed in Table 1. The amplified products were separated on 1.5% agarose gels. The gels were scanned using the Viber Lourmat ECX-F20M system (Vilber Lourmat, France), and proteoglycan genes expression levels were estimated from the intensity of the amplified DNA fragments normalised against the intensity of *GAPDH* using the Gel Analysis program (Russia). 

### 2.5. Immunohistochemistry

Proteoglycan expressions at the protein level were analysed by immunohistochemistry. Briefly, 5-6-*μ*m sections of formalin-fixed, paraffin-embedded tissue sections were deparaffinised, and antigens were retrieved by the treatment with unmasking solution at 95–98°C for 20 min. To block nonspecific staining, the sections were incubated with 10% Fetal Bovine Serum in PBS for 20 min at 37°C. The antibeta actin (1 : 300), antisyndecan-1 (1 : 350), antiglypican-1 (1 : 350), antilumican (1 : 350), antidecorin (1 : 350), anti-CS (1 : 200), and anti-HS (1 : 200) primary antibodies were used for immunostaining (1 h at 37°C). Staining patterns were visualised with TexasRed-conjugated antibody against rabbit IgGs or FITC-conjugated antibody against mouse IgGs (1 : 1000, 30 min at 37°C). The nuclei were counterstained with DAPI and mounted using SlowFade Gold with DAPI mounting medium (Invitrogen, Carlsbad, CA, USA) and observed by fluorescent microscopy (Axio Imager, Carl Zeiss, UK).

### 2.6. Statistical Analysis

Statistical analyses were performed using a computer program ORIGIN Pro 8.0; *P* < 0.05 was considered statistically significant. Data are expressed as means ± SEM.

## 3. Results

Expression of main heparan sulfate proteoglycans (glypican-1, perlecan, and syndecan-1) and chondroitin/dermatan/keratan sulfate proteoglycans (aggrecan, versican, NG2, brevican, decorin, and lumican) was studied in normal, benign, and cancer human prostate tissues by RT-PCR and immunohistochemical analyses. 

### 3.1. Proteoglycans Expression in Normal Human Prostate Tissue

**Figure 1:**
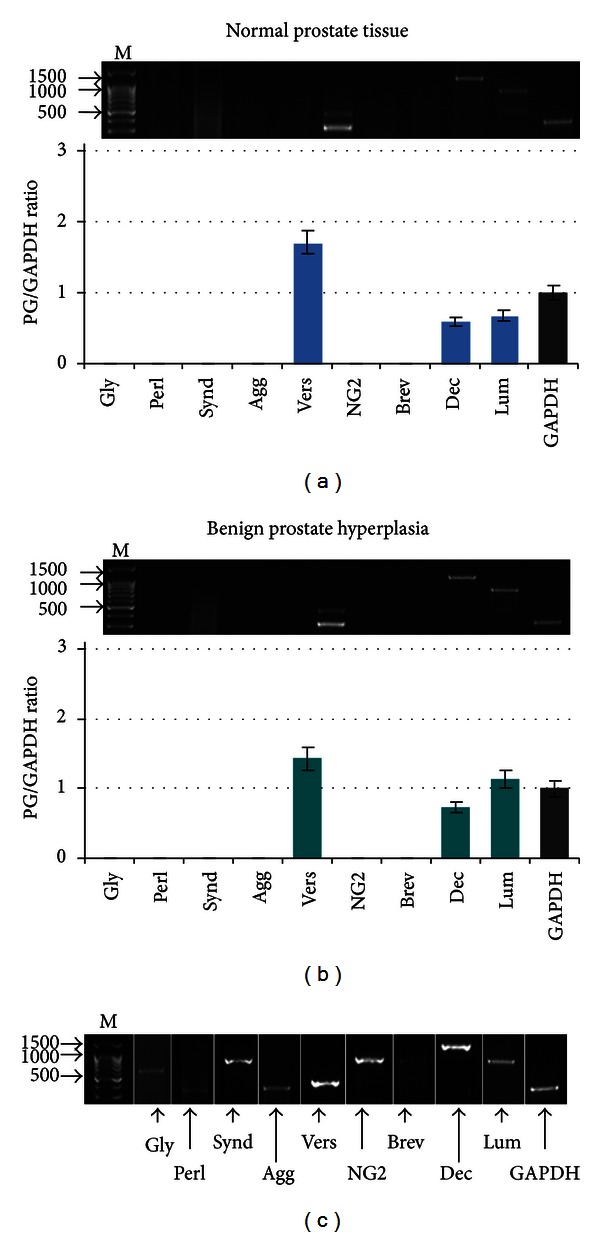
Proteoglycans expression in normal human prostate tissue (a) and benign prostate hyperplasia (b). Intensity of the amplified DNA fragments normalised to that of GAPDH. Representative gels are shown (upper part). The graphs correspond to the lanes order and show the mean expression levels from triplicate experiments (±SD), *P < 0.05 (OriginPro 8.1). (c) Positive controls of undetected proteoglycans. M: 1 Kb-Plus DNA marker (Medigen), Gly: glypican-1, Perl: perlecan, Syn: syndecan-1, Agg: aggrecan, Vers: versican, NG2: neuron-glia 2; Brev: brevican, Dec: decorin, and Lum: lumican.

Relative mRNA levels of the proteoglycans core proteins were determined by multiplex RT-PCR in histologically normal prostate tissues, obtained from the individuals without prostate pathology (Figure 1(a)). 

According to the multiplex RT-PCR data, versican, decorin, and lumican were the main proteoglycans expressed in normal human prostate tissue. The relative mRNA level for each proteoglycan was varied in different tissue samples (versican/GAPDH ratio 0.66–1.71; decorin/GAPDH ratio 0.59–3.25; lumican/GAPDH ratio 0.68–1.18), resulting to the slightly different versican/decorin/lumican ratio in each sample. No glypican-1, syndecan-1, perlecan, or brevican mRNA was detected in the normal prostate tissue by RT-PCR.

In benign prostate pathology (benign prostate hyperplasia (BPH)), the pattern of proteoglycans expression was very similar to that of normal prostate tissue (Figure 1(b)). Versican, decorin, and lumican were the main expressed proteoglycans in BPH as well, with a variation for the relative mRNA levels and ratio of those proteoglycan core proteins (versican/GAPDH ratio 1.11–3.93; decorin/GAPDH ratio 0.73–2.45; lumican/GAPDH ratio 0.80–1.13) similar to that of normal prostate tissue. Additionally, weak expression of glypican-1 and syndecan-1 expression (proteoglycan/GAPDH ratio 0.25–0.57) could be detected in 20–25% of BPH cases. 

### 3.2. Proteoglycans Expression in Human Prostate Tumours

**Figure 2:**
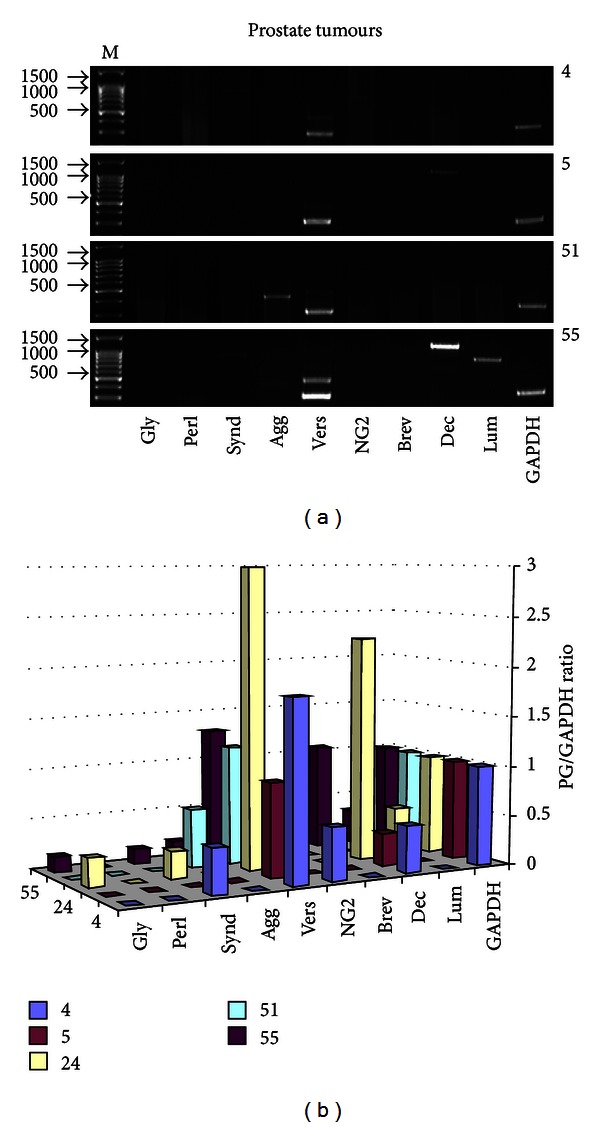
Proteoglycans expression in human prostate tumours. (a) Representative gels are shown. (b) Intensity of the amplified DNA fragments normalised to that of GAPDH. The graphs show the mean expression levels from triplicate experiments (±SD), *P < 0.05 (OriginPro 8.1). 4, 5, 51, and 55 are representative prostate tumours. M: 1 Kb-Plus DNA marker (Medigen).

In prostate tumours, expression of different proteoglycans was changed in different extent, resulting to the individual proteoglycan expression patterns for different clinical samples (Figure 2). 

Few particular expression patterns were detected based on the disappearance of the normally expressed proteoglycans (decorin, lumican) and presence of certain proteoglycan types, usually not detected in normal prostate tissue at mRNA level (glypican-1, syndecan-1, aggrecan, and NG2). 

**Figure 3:**
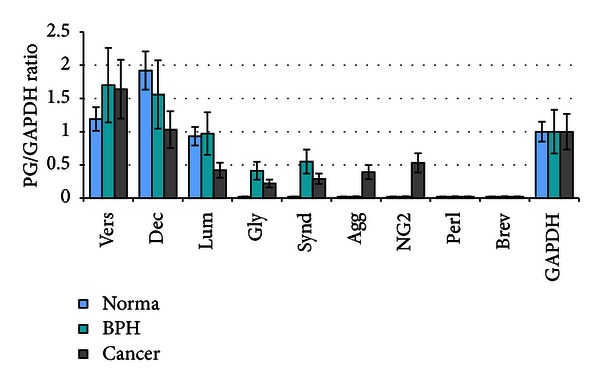
Proteoglycans expression in prostate tumours. Intensity of the amplified DNA fragments normalised to that of GAPDH. The graph shows the mean expression levels from triplicate experiments (±SD), *P < 0.05 (OriginPro 8.1).

However, in spite of highly individual changes in proteoglycans expression patterns in prostate tumours (versican/GAPDH ratio varies between 0.93 and 3.01; decorin/GAPDH ratio 0.33–2.26; lumican/GAPDH ratio 0.34–0.49; absence or presence of glypican-1, syndecan-1, aggrecan, or NG2), some tendencies were outlined from the RT-PCR data (Figure 3). 

Whereas the versican expression level was relatively similar both in normal and malignant prostate tissues (35–40% of increase for versican expression was not considered as significant), decorin and lumican expressions trend towards to be decreased in prostate tumours up to 2-fold. Very careful interpretation is applied in this case because of high variation of decorin and lumican levels in individual tumours, up to complete absence. Additionally, an aggrecan, syndecan-1, and glypican-1 expressions were detected in some prostate tumours. 

As a result, expression patterns for main proteoglycans in prostate tumours were highly individual with an ubiquitous expression for versican and tendency for the decreased decorin and lumican expressions and increased syndecan-1 and glypican-1 expressions in tumour tissues. 

To further study the proteoglycans expression and composition in normal and malignant prostate tissues, immunohistochemical staining for proteoglycans core proteins and polysaccharide GAG chains was conducted.

### 3.3. Immunohistochemical Analysis for Proteoglycans Core Proteins in Normal Prostate Tissue and Prostate Tumours

**Figure 4:**
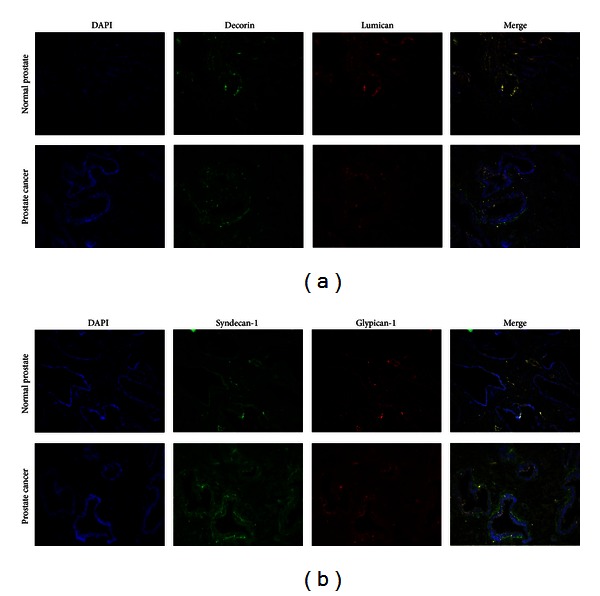
Immunohistochemical analysis of proteoglycan core proteins in normal prostate tissue and prostate tumours. Syndecan-1 and decorin were visualised with FITC-conjugated anti-mouse antibody; glypican-1 and lumican were visualised with TexasRed-conjugated anti-rabbit antibody. The nuclei were counterstained with DAPI. Magnification ×200.

An expression and localisation of the proteoglycans core proteins in the normal and cancer prostate tissues were detected by immunofluorescent staining with the primary antibodies to chondroitin/keratan sulfate proteoglycans decorin and lumican and heparin sulfate proteoglycans syndecan-1 and glypican-1 (Figure 4). 

The results showed that the decorin and lumican proteins were expressed both in normal and tumour prostate tissues at the similar levels and localised mainly in tissue stroma but not in the prostate epithelial cells (Figure 4(a)).

Interesting data were obtained from the immunohistochemical staining for syndecan-1 and glypican-1 core proteins, which clarify the multiplex RT-PCR data on syndecan-1 and glypican-1 expressions in prostate tissues. It was shown that both glypican-1 and syndecan-1 were expressed exclusively in normal prostate epithelial cells with very weak stromal staining for the epitopes (Figure 4(b)). The fact could explain the multiplex RT-PCR results about almost no expression for those proteoglycans at mRNA level, possibly due to minor part of the epithelial cells in total tissue sample. However, in prostate tumours, there was a significant decrease in syndecan-1 and glypican-1 protein expressions in prostate cancer epithelial cells with the simultaneous increase of overall content of the proteoglycans in the stromal compartment of prostate tumour tissue.

Altogether, the results confirm and extend the RT-PCR data on proteoglycans core proteins expression in normal and malignant prostate tissues. 

### 3.4. Immunohistochemical Analysis for Glycosaminoglycans Polysaccharide Chains

Glycosaminoglycans (GAGs) polysaccharide chains are essential part of the matured proteoglycans molecules and principal contributor to the proteoglycans functional properties. 

**Figure 5:**
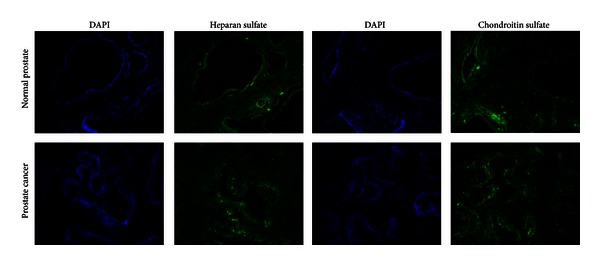
Immunohistochemical analysis of glycosaminoglycans in normal prostate tissue and prostate tumour. Heparan sulfate or chondroitin sulfate chains were stained with the appropriate primary antibodies and visualised with FITC-conjugated anti-mouse antibody. The nuclei were counterstained with DAPI. Magnification ×200.

A presence of appropriate GAGs in the proteoglycans under the study was verified by immunocytochemical staining using antiheparan sulfate and antichondroitin sulfate specific monoclonal antibodies (Figure 5). 

According to the obtained data, heparan sulfates (HS) and chondroitin sulfates (CS) expressions and localisation in normal and cancer prostate tissues were tightly associated with the localisation of corresponding core proteins—HS expression was shifted from the normal prostate epithelial cells to tumour stroma, whereas the CS expression was very similar both in normal and tumour prostate tissues with mainly stromal localisation. 

Taken together, these results show for the first time that normal prostate tissue expresses a specific set of proteoglycans, coming both from prostate epithelial cells (syndecan-1 and glypican-1) and stromal compartment (decorin, lumican, and versican). In prostate tumours, complex changes in proteoglycans occur, with a common trend towards decrease of decorin and lumican expressions, and increase an overall expression of syndecan-1 and glypican-1, shifted from the epithelial cells to tumour stroma. Specific proteoglycans, absent in normal prostate tissue (aggrecan, NG2), were detected in some prostate tumours as well. No perlecan or brevican expression was shown both in normal and cancer prostate tissues. 

## 4. Discussion

Up to date, there are not so many data about the expression of different proteoglycans and their interrelations in prostate cancer [5]. 

The first key result of the present study is an expression pattern of proteoglycans in normal human prostate tissue, as a starting point for the consequent investigation of the proteoglycans expression changes in prostate cancer. Earlier, versican [19], decorin [8], lumican [20, 21], and syndecan-1 [16, 22] expression in normal human prostate tissues has been shown in different experiments. Our results showed the expressions of all the proteoglycans as well and extended the list with glypican-1 expression in prostate epithelial cells and tumour stroma (Figures 1 and 4).

In prostate tumours, no definite “prostate cancer” pattern was shown for the proteoglycans expression. Few different patterns were identified, with significant variation in expression level for each proteoglycan from high expression up to complete disappearance. In such a case, it was complicated to perform a real statistical analysis, and all the “means” are very relative. It is a reason why we operate only with tendencies or trends in the analysis of the obtained data.

According our results, versican was the most stably expressed extracellular proteoglycan in prostate tumours, with the mRNA levels similar to that in normal prostate tissue (Figure 3). It is slightly controversial with the published data on elevated levels of versican protein in prostate cancer, associated with disease progression in early-stage prostate cancer [6, 7]. Possibly, an accumulation of versican in cancer prostate tissue is due to either posttranscriptional activation of versican expression or decreased versican degradation in prostate tumours but not versican regulation at mRNA level.

On decorin expression in prostate cancer, two controversial results were published earlier. It was shown that decorin concentration is increased in the prostatic tissue of men with early-stage prostate cancer [7] or reduced in prostate cancer stroma compared to nonmalignant prostate stroma [8]. Our results outlined a tendency for the decreased decorin expression in prostate tumours (Figure 3); however, a significant individual variation of decorin mRNA levels in different prostate tumours could explain the discrepancy of the experimental data from different sources. 

Along with versican and decorin, we identified lumican as a most ubiquitously expressed proteoglycan in prostate tissues with the similar expression levels in normal and pathological tissues. Earlier, the only published paper showed lumican upregulation in BPH when compared with normal prostate tissues [20], with no data for lumican expression in prostate tumours.

Glypican-1 is an another proteoglycan, which expression was detected in prostate cancer for the first time. Interestingly, in normal prostate tissue, only epithelial cells expressed glypican-1, whereas prostate tumours displayed significant decrease of glypican-1 expression in cancer epithelial cells and an elevated glypican-1 levels in tumour stroma (Figure 4).

A similar effect was shown for the syndecan-1 expression change in prostate tumours. Syndecan-1 expression was significantly decreased in the cancer epithelial cells but increased in tumour stroma (Figure 4). It was not known for prostate cancer, although was shown for some other cancers. For example, syndecan-1 expression was found mainly in epithelial cells and reduced during malignant transformation of various epithelia, and this loss correlated with the histological differentiation grade of squamous cell carcinomas of the head and neck [22]. The loss of epithelial syndecan-1 and strong stromal syndecan-1 was associated with an unfavorable prognosis in gastric cancer [23].

These results suggest a hypothesis for the controversial data on syndecan-1 expression in prostate cancer. Analysing the literature, one could mention that almost all data on the decreased expression of syndecan-1 were obtained from the cell culture experiments *in vitro*, based on the prostate cancer cell lines of epithelial origin [15, 16]. However, most of the results on the increased expression of syndecan-1 in prostate tumours were shown by immunohistochemistry [12–14]. Possibly, data on simultaneous disappearance of syndecan-1 from prostate cancer epithelial cells and overall increase of syndecan-1 content in tumour stroma could contribute to the understanding of the functional role of syndecan-1 in prostate carcinogenesis. 

Totally, our results are in a good agreement with the published data on the proteoglycans expression in prostate cancer and, for the first time, show a common patterns for proteoglycans expression in the normal and tumour prostate tissues.

## 5. Conclusions

Taken together, the results of the present study show that

 (i) normal human prostate tissue expresses a specific set of proteoglycans, localised both in prostate epithelial cells (syndecan-1, glypican-1) and in the tissue stroma (versican, decorin, and lumican);
 (ii) in prostate tumours, proteoglycans pattern is completely changed due to decreased expression of stromal proteoglycans (decorin, lumican), increased expression of syndecan-1 and glypican-1 and their relocalisation to tumour stroma, and appearance of additional proteoglycans like aggrecan and NG2;
 (iii) highly individual patterns for proteoglycans expression are shown in prostate tumours due to various combinations of the expressed proteoglycans and their expression levels.

## Figures and Tables

**Table 1:** Primer sequences and PCR conditions.

Gene	Sequences	DNA fragment, bp	PCR conditions	
			Annealing T, °C	Cycles
Gly	F 5′-GAGCTGCGGCGAGGTCCG-3′	648	55	33
	R 5′-CTGGTCTACTGTGCTCACTGCC-3′			
Perl	F 5′-TCCCTGGACACAGATGGC-3′	314	55	35
	R 5′-ACCCATGCAGAAACAGGG-3′			
Synd	F 5′-GCCCCCTGAAGATCAAGATGG C-3′	790	61	30
	R 5′-CCTCCTGTT TGGTGGGCTTCTG-3′			
Agg	F 5′-CTT CTA CCG CCC CAC TGG CC-3′	400	63	33
	R 5′-GCCAGCCGGCGTCACACTG-3′			
Vers	F 5′-GGCCAGCCCCCTGTTGTAGA-3′	308	63	33
	R 5′-AGGGATCAGCGCCTCGACTC-3′			
NG2	F 5′-AGCCCTTTTGGGAGGCCCATG-3′	758	61	30
	R 5′-GAAGATGCCTGCCACGCTGC-3′			
Brev	F 5′-GCCCTCACCATCCCTTGCCA-3′	476	61	30
	R 5′-TCCGACAGCCAGCCAGCATC-3′			
Dec	F 5′-GGCCACTATCATCCTCCTTCTGC-3′	1031	59	25
	R 5′-ATGGCAGAGCGCACGTAGACAC-3′			
Lum	F 5′-CTCTCTTCCTGGCATTGATTGGTGG-3′	720	61	30
	R 5′-GACAGATCCAGCTCAACCAGGG-3′			
GAPDH	F 5′-GGGCGCCTGGTCACCAG-3′	350	59	22
	R 5′-AACATGGGGGCATCAGCAGAG-3′			

## Abbreviations

BPH: Benign prostate hyperplasia
PG: Proteoglycan
GAG: Glycosaminoglycan
ECM: Extracellular matrix
HS: Heparan sulfate
CS: Chondroitin sulfate
DS: Dermatan sulfate
Gly: Glypican-1
Perl: Perlecan
Syn: Syndecan-1
Agg: Aggrecan
Vers: Versican
NG2: Neuron-glia 2
Brev: Brevican
Dec: Decorin
Lum: Lumican.
